# Overdiagnosis of Melanoma In Situ

**DOI:** 10.3390/jcm15114339

**Published:** 2026-06-03

**Authors:** Maria Elisabetta Greco, Antonio Di Guardo, Annunziata Dattola, Giovanni Pellacani

**Affiliations:** 1 Dermatology Clinic, Department of Medical and Cardiovascular Sciences, University of Rome “Sapienza”, 00185 Rome, Italy; antonio.diguardo@uniroma1.it (A.D.G.); nancydattola@gmail.com (A.D.); giovanni.pellacani@uniroma1.it (G.P.); 2 Istituto Dermopatico dell’Immacolata (IDI-IRCCS), 00167 Rome, Italy

**Keywords:** melanoma in situ, overdiagnosis, screening, overdetection

## Abstract

**Background/Objectives:** The incidence of melanoma in situ (MIS) has increased markedly in several high-income countries, often outpacing invasive melanoma without a proportional rise in mortality. This divergence has raised concerns regarding overdiagnosis, defined as the detection of biologically indolent disease that would not have caused harm if left untreated. This review aims to synthesize contemporary evidence on the overdiagnosis of MIS, focusing on epidemiological patterns, screening-related detection, pathological interpretation, quantitative estimates, and clinical consequences. **Methods:** A narrative review with systematic search elements was conducted using a structured approach informed by PRISMA principles. PubMed was searched for studies published between January 2017 and March 2026, and eligible studies were selected based on relevance to melanoma overdiagnosis, MIS incidence, screening, and diagnostic variability. **Results:** The literature consistently demonstrates that increases in melanoma incidence are largely driven by MIS and thin invasive lesions rather than advanced disease. Screening and surveillance preferentially increase detection of low-risk lesions, while variability in dermatopathological interpretation contributes to diagnostic drift. Quantitative studies suggest that a substantial proportion of MIS diagnoses may represent overdiagnosis, exceeding 60–80% in some settings. All of this entails significant direct and indirect social costs in several countries. **Conclusions:** Overdiagnosis of MIS is a significant and multifactorial phenomenon. MIS represents a heterogeneous entity, and future strategies should focus on improving diagnostic precision, risk stratification, and minimizing harm associated with unnecessary diagnosis.

## 1. Introduction

Overdiagnosis is defined as the detection of disease that would not have caused symptoms, morbidity, or mortality during a patient’s lifetime. In melanoma, concern about overdiagnosis has grown in parallel with the observation that incidence rates have increased substantially over recent decades, particularly for early-stage disease, whereas mortality has remained relatively stable or has changed only modestly [1]. This discrepancy has prompted reconsideration of the biological significance of lesions currently classified as melanoma, especially melanoma in situ (MIS).

MIS occupies a conceptual and biological boundary between benign melanocytic proliferations and invasive melanoma. While some lesions classified as MIS likely represent true precursors to invasive disease, accumulating evidence suggests that a proportion may have limited malignant potential and may never progress if left untreated [2,3]. This heterogeneity complicates both clinical management and epidemiological interpretation.

The increasing incidence of MIS appears to be driven by multiple interacting factors, including greater public awareness, increased skin surveillance, widespread use of dermoscopy, lower thresholds for biopsy, and evolving histopathological criteria. These elements collectively contribute to both increased detection of lesions and shifts in diagnostic classification, thereby amplifying the phenomenon of overdiagnosis [2,4].

## 2. Materials and Methods

This narrative review with systematic search elements was conducted using a structured methodology informed by the principles of the Preferred Reporting Items for Systematic Reviews and Meta-Analyses (PRISMA), adapted to the context of a non-systematic synthesis. A comprehensive literature search was performed using the PubMed database to identify relevant publications addressing overdiagnosis of melanoma in situ.

The search covered the period from January 2017 to March 2026. Search terms included combinations of “melanoma in situ,” “in situ melanoma,” and “stage 0 melanoma” with “overdiagnosis,” “overdetection,” “diagnostic drift,” “screening,” and “early detection.” Additional targeted searches were conducted using related terms such as “thin melanoma,” “melanocytic lesions,” and “dermatopathology variability.” Reference lists of included studies were manually screened to identify further relevant publications.

Studies were considered eligible if they were indexed in PubMed, published in English, and addressed melanoma overdiagnosis, MIS epidemiology, screening, pathology, or diagnostic variability. Eligible study designs included observational studies, registry-based analyses, ecological studies, scoping reviews, and influential commentaries. Case reports, small case series, non-human studies, and articles not directly addressing overdiagnosis were excluded.

Titles and abstracts were screened for relevance, followed by full-text assessment of selected articles. Emphasis was placed on studies examining epidemiological trends, screening effects, diagnostic variability, quantitative estimates of overdiagnosis, and clinical or psychosocial outcomes. Although formal duplicate screening was not performed, priority was given to methodologically robust and high-impact studies.

Due to heterogeneity in study design and definitions of overdiagnosis, a quantitative meta-analysis was not feasible. Instead, findings were synthesized qualitatively, with emphasis on identifying consistent patterns across different domains of evidence. The study selection process followed a PRISMA-like flow, with approximately 180 records initially identified, 120 screened, 60 assessed in full text, and 17 included in the final synthesis.

From the initially identified records, studies were selected through a stepwise screening process based on predefined relevance criteria. Inclusion was restricted to studies directly addressing overdiagnosis or providing indirect epidemiological, screening-related, or diagnostic evidence relevant to melanoma in situ. Priority was given to methodologically robust, high-impact, and non-redundant studies, while overlapping or less informative reports were excluded. Given the heterogeneity of study designs and definitions, a purposive selection approach was adopted to ensure a balanced and conceptually coherent synthesis rather than exhaustive inclusion.

### AI Statement

The preparation of this work involved the use of generative artificial intelligence tools solely as support for language revision, content organization, and improvement of clarity and readability. The scientific content, selection of bibliographic sources, critical interpretation of data, and final revision remain the sole responsibility of the author.

## 3. Results

Epidemiological data provide strong support for the presence of overdiagnosis in MIS. One of the most compelling observations is the divergence between increasing incidence and relatively stable mortality. Olsen and colleagues demonstrated that the ratio of in situ to invasive melanoma has increased substantially over time across multiple populations, including Queensland, the United States, and Scotland [5]. Notably, the age distribution of MIS was similar to or older than that of invasive melanoma, which is inconsistent with the expectation that MIS represents an obligate precursor detected earlier in its natural history.

A further indication of the rising incidence of melanoma in situ comes from the population-based study by Thomas et al., which analyzed data from the Icelandic Cancer Registry over the period 1957–2021. The authors found a significant increase in melanoma in situ incidence in both sexes, with rates rising from 0.2 to 4.0 cases per 100,000 person-years in men and from 0.9 to 9.5 cases per 100,000 person-years in women, showing a statistically significant linear trend (*p* = 0.001). In particular, among women, the age-standardized incidence of melanoma in situ peaked at 12.4 per 100,000 person-years between 1997 and 2001, subsequently declined to 4.2, and then showed a renewed upward trend in the most recent period (2017–2021), with an annual percent change (APC) of 1.43 [6].

Additional evidence supporting the possibility that the rising incidence of melanoma, particularly early-stage lesions, may partly reflect increased diagnostic scrutiny rather than a true rise in lethal disease comes from the Danish registry-based study by Nielsen et al. Analyzing national pathology and mortality data from 1999 to 2019, the authors observed a marked increase in the incidence of melanoma in situ, which rose by 476% in men and 357% in women, alongside an even more pronounced increase in atypical melanocytic lesions. Over the same period, invasive melanoma incidence also increased, especially between 1999 and 2011, whereas melanoma-specific mortality remained essentially stable, with no significant temporal trend in either sex. Notably, the rise in melanoma incidence paralleled a substantial increase in biopsy rates, suggesting that intensified diagnostic activity may have contributed to the growing number of detected lesions [7].

These findings are supported by broader analyses. Welch and colleagues showed that the rise in melanoma incidence in the United States has been largely driven by localized disease, suggesting that increased detection rather than a true increase in biologically aggressive disease accounts for much of the observed trend [1]. A recent scoping review further confirmed that increases in melanoma incidence are not matched by comparable increases in mortality, and that this discrepancy is most pronounced for MIS and thin melanomas [8].

Country-specific studies reinforce these observations. In Italy, registry-based analyses have shown that the increase in melanoma incidence is particularly pronounced for MIS and lesions less than 0.8 mm in thickness, while mortality trends remain relatively stable [9]. These data collectively support the interpretation that overdiagnosis is a major contributor to the epidemiology of MIS.

The following table summarizes the principal epidemiological studies supporting a disproportionate increase in melanoma in situ diagnoses, often in the absence of a corresponding rise in melanoma-specific mortality, thereby reinforcing the hypothesis that overdiagnosis contributes substantially to current MIS epidemiology (Table 1).

### 3.1. Screening and Overdetection

Evidence from screening studies indicates that increased surveillance contributes significantly to the detection of MIS. In the QSkin cohort study, Whiteman and colleagues demonstrated that individuals who underwent skin examinations had higher subsequent melanoma detection rates, particularly for in situ lesions, along with increased biopsy rates [11]. These findings suggest that screening preferentially identifies lesions that may not have clinical significance.

Similarly, a large US screening initiative reported increased detection of thin melanomas without a corresponding reduction in the incidence of thicker, more advanced disease [12]. This pattern is consistent with overdetection rather than effective early detection of clinically relevant cancers.

Technological advances such as dermoscopy may further contribute to this phenomenon. While dermoscopy improves sensitivity for detecting melanocytic lesions, it may also increase the detection of subtle lesions with uncertain biological potential, thereby contributing to overdiagnosis when specificity is insufficient [4].

Moreover, the introduction of advanced imaging technologies, including 3D total body photography, sequential dermoscopy imaging, and artificial intelligence, has likely contributed to the increasing detection of melanoma at earlier stages, particularly melanoma in situ. These tools improve surveillance in high-risk individuals by enabling more accurate identification of subtle morphological changes over time and facilitating the recognition of lesions that might otherwise remain unnoticed. In particular, 3D total body photography allows comprehensive longitudinal monitoring of the entire skin surface, while sequential dermoscopy enhances the assessment of equivocal melanocytic lesions. Artificial intelligence may further increase diagnostic sensitivity, especially in the evaluation of early and clinically subtle lesions [13,14].

This pattern may reflect a paradox whereby improved early detection increases the apparent incidence of melanoma, while many of the additional cases identified are low-risk and do not translate into a corresponding reduction in mortality.

### 3.2. Pathology and Diagnostic Drift

Overdiagnosis of MIS is also influenced by changes in diagnostic criteria, a phenomenon often referred to as overdefinition. Over time, histopathological thresholds for classifying melanocytic lesions have evolved, resulting in an increased tendency to diagnose borderline lesions as MIS [2,3].

Empirical studies support the existence of diagnostic variability. Nearly half of dermatopathologists surveyed consider MIS to be overdiagnosed as a public health issue [15]. Furthermore, specialist training in dermatopathology has been associated with a greater likelihood of assigning higher-grade diagnoses to the same lesions, suggesting that interpretative factors contribute to incidence trends [16]. One particularly significant factor is the role of specialist training. Kerr et al. have shown that dermatopathologists tend to assign higher-grade diagnoses than general pathologists when interpreting the same melanocytic lesions. In particular, dermatopathologists more frequently diagnose early-stage invasive melanomas (pT1a), whilst general pathologists tend to classify the same lesions as atypical nevi [16]. Furthermore, among dermatopathologists themselves, a higher caseload of melanocytic lesions is associated with more severe diagnoses [16] This suggests that greater exposure and specialization may, paradoxically, lower the diagnostic threshold, likely through mechanisms such as heightened sensitivity to histopathological patterns and medico-legal pressures.

Another point worth considering is inter-observer variability. The largest study on diagnostic reproducibility revealed that on average 10 different diagnostic terms were applied to each case [17]. Concordance rates with the reference diagnosis were particularly low in the intermediate spectrum as moderately dysplastic nevi: 25% concordance; severely dysplastic nevi/MIS: 40% concordance; invasive melanoma pT1a: 43% concordance; benign nevi: 92% concordance; invasive melanoma ≥ pT1b: 72% concordance [17].

Haggenmüller et al. (2025) confirmed these findings in a prospective multicenter German context: among 792 suspicious lesions, complete agreement among eight expert pathologists was achieved in only 53.5% of cases, with particularly marked discordance for non-invasive melanomas (complete agreement in only 10 of 73 cases) [18]. Alexander et al. (2026) reported an overall diagnostic discordance of 33% among 1491 lesions, with indeterminate-risk lesions showing 79.2% discordance, and 72.1% of discordant cases being downgraded to benign entities upon review [19].

Reproducibility studies therefore further highlight the inconsistency of diagnoses in the borderline area between benign nevi and melanoma. These findings suggest that some MIS diagnoses may reflect variability in classification rather than actual biological differences.

### 3.3. Quantitative Estimates of Overdiagnosis

Recent research has attempted to quantify the extent of melanoma overdiagnosis. In an ecological analysis of US data, Adamson and colleagues estimated that a substantial proportion of melanoma cases represent overdiagnosis, with particularly high proportions for MIS, exceeding 80% in some subgroups [19].

Australian studies have produced similar findings. Lindsay and colleagues estimated that between 67% and 76% of stage 0 melanomas are overdiagnosed, representing a large number of excess diagnoses and substantial healthcare costs [20]. These estimates are consistent with findings from broader reviews, which report overdiagnosis rates ranging from approximately 29% to 60% across melanoma overall, with higher values for MIS [8].

### 3.4. Clinical and Societal Impact

Although MIS is associated with an excellent prognosis following excision, its diagnosis carries important consequences. Management typically involves surgical treatment, follow-up visits, and ongoing surveillance, all of which contribute to healthcare utilization and costs [20].

The economic burden associated with the overdiagnosis of melanoma in situ (MIS) has been most comprehensively quantified in Australia, where estimates suggest annual direct healthcare costs of approximately AUD $17.7–18.6 million (USD $12–13 million) within the first year following diagnosis DA 16 A 30 [20,21]. Although global cost estimates remain unavailable, data from individual countries provide insight into the magnitude of this issue.

### 3.5. Australia: Detailed Cost Estimates

The most robust analysis to date was conducted by Lindsay et al. (2024), who estimated that, in Australia in 2021, between 71% and 76% of MIS cases were overdiagnosed, corresponding to approximately 19,829–20,811 cases [20]. The associated annual direct healthcare costs ranged from AUD $17.7 million (95% CI 17.4–17.9 million) to AUD $18.6 million (95% CI 18.3–18.8 million) in the year following diagnosis [20]. When thin invasive melanomas (stage IA) are included, the estimated total cost of overdiagnosis increases to approximately AUD $20.2–21.4 million annually [20]. Importantly, these estimates account only for direct medical costs incurred during the first year after diagnosis and do not include long-term surveillance, indirect costs such as productivity losses, or the psychological burden experienced by patients [20,22].

### 3.6. United States: Incidence and Treatment Costs

In the United States, the projected incidence of MIS for 2025 is approximately 107,240 cases [23]. While comprehensive national estimates of the cost of MIS overdiagnosis are lacking, available data on treatment costs provide useful context. The mean cost of MIS treated with wide local excision by dermatologists is approximately $1089 (95% CI $941–1237), compared with $5172 (95% CI $2419–7925) when managed by other surgical specialties [24]. Mohs micrographic surgery performed by dermatologists has a mean cost of $2325 (95% CI $2241–2409) [24]. Assuming that approximately 60% of MIS cases represent overdiagnosis, consistent with epidemiological estimates, and applying an average treatment cost of $1500–2000 per case, the annual direct cost of MIS overdiagnosis in the United States may exceed $100 million, excluding follow-up and surveillance costs [23,25].

### 3.7. Italy: Stage-Specific Cost Data

An Italian cost analysis reported a mean per-patient cost of €149 for stage 0 melanoma (MIS), compared with €66,950 for stage IV disease [26]. Although this study did not specifically address overdiagnosis, it highlights that even early-stage melanoma is associated with measurable healthcare expenditure, which becomes substantial when multiplied across large populations.

### 3.8. Global Trends and Projections

Globally, melanoma incidence is projected to increase from 331,722 cases in 2022 to approximately 510,000 cases annually by 2040 [23]. The proportion of MIS among all melanoma diagnoses has been rising in most high-income countries, particularly in populations with predominantly fair skin, including Australia, Europe, and North America [23,27]. In England, for example, 86,792 MIS cases were recorded between 2001 and 2020, with incidence rates increasing annually by 7.51% between 2001 and 2015, followed by a slower increase of 1.90% between 2015 and 2019 [28].

### 3.9. Indirect and Societal Costs

Existing cost estimates substantially underestimate the true economic burden, as they typically include only direct healthcare expenditures. Additional costs include productivity losses due to time off work for diagnostic and therapeutic procedures, patient travel and time costs, and long-term surveillance, which may extend for at least five years or even lifelong [29]. Furthermore, the psychological impact of a melanoma diagnosis is considerable: patients diagnosed with MIS may experience levels of anxiety and fear of recurrence comparable to those with invasive melanoma [22]. A systematic review has also demonstrated that productivity losses associated with melanoma morbidity and premature mortality are significant, with mean years of life lost due to metastatic melanoma estimated at 20.4 years, exceeding those observed for several other cancers (16.2 years) [30].

Several studies have evaluated the cost-effectiveness of melanoma screening strategies. Population-based screening in Belgium has been estimated to cost between €18,687 and €33,072 per quality-adjusted life year (QALY) gained, suggesting moderate cost-effectiveness [24]. In the United States, biennial screening of white men aged 50–75 years has been estimated at $26,503 per QALY when initiated at age 60, increasing to $67,970 per QALY when initiated at age 50 [31]. However, these models typically assume that screening reduces the incidence of advanced melanoma, an assumption that may be increasingly challenged in the context of improved systemic therapies [32].

The economic implications of MIS overdiagnosis extend beyond direct treatment costs and raise important considerations for healthcare policy. Recent clinical guidelines, such as those from the National Comprehensive Cancer Network, acknowledge that management of MIS may include not only surgical excision but also topical therapies or active surveillance, particularly in patients with limited life expectancy or significant comorbidities [29]. This reflects a growing recognition that not all MIS lesions require immediate intervention and that more conservative approaches may reduce unnecessary healthcare utilization.

Overall, these findings underscore the need for strategies aimed at reducing overdiagnosis, including refinement of diagnostic criteria, implementation of risk-stratified screening approaches, and reconsideration of disease nomenclature to mitigate both the psychological and economic consequences of labeling indolent lesions as cancer.

## 4. Discussion

The available evidence indicates that overdiagnosis of MIS is substantial and multifactorial. The phenomenon arises from the interaction of increased detection through screening and surveillance, advancements in diagnostic technologies, and evolving pathological criteria that expand disease definitions.

Importantly, MIS should not be regarded as a uniform entity. Instead, it encompasses a spectrum of lesions with varying malignant potential, ranging from true precursors of invasive melanoma to biologically indolent proliferations. The inability to reliably distinguish between these categories remains a central challenge in melanoma research and clinical practice.

A further point that deserves emphasis is the persistent dissociation observed in many settings between the rising incidence of early melanoma diagnoses, particularly MIS, and the relatively stable trends in melanoma-specific mortality reported over time. Although this pattern cannot be interpreted as direct proof of overdiagnosis, it raises important questions about the biological significance of at least a proportion of lesions currently classified as MIS.

If all lesions diagnosed at this stage represented obligate precursors of invasive and potentially lethal melanoma, one would expect that their increasing detection and excision would be followed by a more consistent reduction in advanced disease and melanoma mortality. The fact that this relationship appears less straightforward suggests that some diagnosed lesions may have limited or no capacity for clinically meaningful progression.

This interpretation is also supported by the broader concept that increased diagnostic intensity can reshape the apparent epidemiology of melanoma without necessarily changing the burden of life-threatening disease.

Greater surveillance, lower thresholds for biopsy, wider use of dermoscopy, digital monitoring, and heightened awareness among both clinicians and patients all contribute to the identification of increasingly subtle melanocytic proliferations. While these developments have undoubtedly improved the recognition of truly dangerous lesions, they have also expanded the pool of lesions entering the diagnostic pathway.

In this context, the growing detection of borderline or equivocal lesions may inflate MIS incidence, particularly when pathological criteria favour caution and classify ambiguous lesions within the malignant spectrum.

In addition, emerging diagnostic technologies, including artificial intelligence, reflectance confocal microscopy, and line-field optical coherence tomography, have demonstrated increased sensitivity for the detection of early and subclinical melanocytic lesions. Although these tools improve diagnostic accuracy, their widespread adoption may contribute to a stage shift toward melanoma in situ and very early disease. However, direct population-level evidence linking these technologies to increased melanoma in situ incidence or reduced mortality remains limited.

Another relevant issue concerns the progressive lowering of diagnostic thresholds in dermatopathology. Over time, the histopathological interpretation of melanocytic lesions has been influenced by greater attention to architectural disorder, pagetoid spread, cytologic atypia, and other features that may overlap between reactive, dysplastic, and early malignant proliferations. As a result, lesions that might previously have been regarded as severely atypical or of uncertain malignant potential are now more readily categorized as MIS. This diagnostic drift does not imply error in the conventional sense but rather reflects the intrinsic subjectivity of current criteria and the tendency of modern medicine to prioritize sensitivity over specificity.

In melanoma, however, this approach has important consequences, as even a histologically correct diagnosis may not necessarily correspond to a lesion destined to become clinically harmful. The heterogeneity of MIS therefore remains a central conceptual problem. Some lesions likely represent genuine intraepidermal melanoma with the potential to evolve into invasive disease if left untreated, whereas others may remain stable for prolonged periods, progress extremely slowly, or perhaps never acquire invasive capability.

At present, routine clinical and pathological assessment does not allow reliable stratification between these biologically distinct subsets. This limitation has major implications not only for epidemiologic interpretation, but also for patient care, because it means that all lesions are generally managed according to a worst-case model despite likely differences in natural history.

From a clinical perspective, the consequences of overdiagnosis should not be underestimated. Even when treatment is relatively minor, a melanoma diagnosis may carry substantial psychological burden, including anxiety, altered self-perception, repeated follow-up visits, and concern regarding recurrence or familial risk. In addition, diagnosis often triggers broader surveillance strategies, further biopsies, and repeated excisions of additional suspicious lesions, creating a cascade of interventions that may not always translate into meaningful health benefit. The potential harms are therefore not limited to surgical morbidity or healthcare costs but also include the long-term emotional and behavioural effects of labelling patients with a malignant diagnosis.

These considerations do not argue against early detection itself but rather support a more nuanced approach to the detection and classification of very early melanocytic lesions. The challenge is not simply to find melanoma earlier, but to identify which lesions truly require intervention and which represent indolent disease within an increasingly sensitive diagnostic system.

In this regard, future progress will likely depend on integrating conventional histopathology with ancillary tools capable of improving biological risk stratification. Molecular markers, genomic profiling, artificial intelligence-assisted image analysis, and clinicopathologic prediction models may eventually help distinguish lesions with true invasive potential from those unlikely to cause harm. Equally important is the need to refine terminology and disease framing. The label “melanoma in situ” may imply an inevitable trajectory toward invasive melanoma, a concept that is not yet fully supported for all lesions included under this category. More precise subclassification systems, possibly incorporating degrees of certainty or biologic risk, may reduce the binary distinction between benign and malignant that currently characterizes many melanocytic diagnoses. Such refinement could improve both diagnostic reproducibility and the alignment between pathological nomenclature and biological behaviour.

Finally, the issue of MIS overdiagnosis should be viewed within the broader debate on cancer overdiagnosis across multiple specialties. As in other fields, advances in detection have outpaced our ability to discriminate aggressive from indolent disease. In melanoma, this imbalance is especially relevant because visual screening is widespread, skin biopsies are easily performed, and the threshold for excision is understandably low in the face of a potentially lethal cancer. The result is a diagnostic environment in which caution favours early identification but may also increase the detection of lesions with uncertain clinical relevance. Recognizing this tension is essential for developing more balanced strategies that preserve the benefits of melanoma prevention while minimizing unnecessary diagnosis and treatment [33].

Future strategies should therefore not aim to reduce diagnostic accuracy, but to improve diagnostic proportionality. A universal, highly intensive screening approach may amplify the detection of low-risk lesions in individuals with limited absolute risk, potentially increasing biopsies, excisions, psychological burden, and healthcare costs without clear evidence of a proportional mortality benefit. Current evidence on routine skin examinations remains mixed, with earlier detection not consistently translating into reduced melanoma-specific mortality [34].

A more balanced strategy involves risk-tailored surveillance, in which screening intensity is calibrated according to individual risk. Factors such as personal history of melanoma, family history, nevus burden and phenotype, age, skin phototype, history of severe sunburn, immunosuppression, genetic predisposition, and cumulative ultraviolet exposure should guide follow-up strategies. Emerging evidence supports the feasibility and potential value of such risk-stratified approaches, particularly in high-incidence settings [11,35].

Family history is a particularly important determinant, as melanoma risk is significantly increased among individuals with affected first- or second-degree relatives, especially when diagnoses occur at younger ages. Likewise, patients with a prior melanoma represent a well-established high-risk group and may benefit from more intensive and structured surveillance compared with the general population [36].

Advanced diagnostic technologies, including dermoscopy, total body photography, reflectance confocal microscopy, LC-OCT, and artificial intelligence–based tools, should be integrated selectively rather than indiscriminately. While these modalities enhance diagnostic sensitivity and may improve the evaluation of equivocal lesions, their widespread use without appropriate risk stratification may contribute to a diagnostic shift toward increasingly subtle or biologically indolent lesions. Their optimal role lies not simply in increasing detection, but in refining diagnostic specificity and supporting clinical decision-making. Current European melanoma guidelines support the use of advanced imaging techniques, such as confocal microscopy, in selected diagnostic contexts rather than as universal screening tools [37,38].

From this perspective, limiting MIS overdiagnosis will require a multidimensional strategy, including improved risk prediction models, more selective screening practices, greater standardization in dermatopathological interpretation, and clearer communication regarding the biological heterogeneity of MIS. Importantly, future research should focus not only on detection rates but also on downstream outcomes such as invasive melanoma incidence, melanoma-specific mortality, quality of life, and the burden of overtreatment. The challenge is not to reduce early detection, but to make it more precise, individualized, and clinically meaningful [1,33,39].

## 5. Conclusions

The contemporary literature provides strong evidence that overdiagnosis of melanoma in situ is a significant issue in modern dermatology. The disproportionate increase in MIS incidence relative to invasive melanoma, combined with evidence from screening studies, pathology research, and quantitative analyses, supports the conclusion that many MIS diagnoses may not represent clinically meaningful disease.

At the same time, MIS remains a heterogeneous category, and not all lesions are biologically trivial. The key challenge moving forward is to distinguish lesions that require intervention from those that can be safely managed conservatively, thereby reducing the harms associated with overdiagnosis while preserving the benefits of early detection.

Future efforts should focus on improving the specificity of early detection strategies, refining pathological classification systems, and developing biomarkers capable of predicting lesion behavior. Enhanced patient communication is also essential to ensure that the risks and implications of diagnosis are clearly understood. Further large-scale, prospective studies are warranted to clarify the natural history of melanoma in situ, improve risk stratification, and identify reliable markers capable of distinguishing clinically significant lesions from biologically indolent disease.

## Figures and Tables

**Table 1:** Summary of the main epidemiological studies reporting an increase in melanoma in situ (MIS) diagnosis across different populations.

Study	Country/Setting	Period	Quantitative Data on MIS Increase	Other Key Numerical Findings
Olsen et al., 2022 [5]	Queensland/US White population/Scotland	1982–2018	MIS: invasive melanoma incidence ratio increased from <0.3 (1982) to 1.95 (Queensland), 0.93 (US Whites), 0.58 (Scotland) in 2018	Mean age at MIS diagnosis was similar to or older than invasive melanoma
Thomas et al., 2025 [6]	Iceland	1957–2021	MIS incidence increased from 0.2 → 4.0/100,000 person-years in men and 0.9 → 9.5/100,000 person-years in women	Women: MIS peak 12.4/100,000 (1997–2001), then 4.2, then renewed increase in 2017–2021; APC 1.43
Nielsen et al., 2024 [7]	Denmark	1999–2019	MIS incidence increased by +476% in men and +357% in women	MIS incidence: 5.12 → 29.48 in men; 7.26 → 33.21 in women. Biopsy rate: +153% men, +118% women (to 2011). Mortality: no significant trend
Bjørch et al., 2024 [8]	Scoping review	Multiple studies	Increase mainly involved MIS and thin melanomas	Estimated overdiagnosis across included studies: 29–60%
Bucchi et al., 2023 [9]	Italy (Emilia-Romagna)	Registry study	Steepest increase for MIS: EAAPC 10.2 in men, 6.9 in women	Melanomas < 0.8 mm: EAAPC 9.1 in men, 5.2 in women; mortality comparatively stable
Adamson et al., 2024 [10]	United States	1975–2018	Lifetime risk of MIS increased from 0.17% → 2.7% in white men and 0.08% → 2.0% in white women	Estimated overdiagnosed MIS in 2018: 89.4% in white men and 85.4% in white women

## Data Availability

No new data were created.
